# Longitudinal multi-centre brain imaging studies: guidelines and practical tips for accurate and reproducible imaging endpoints and data sharing

**DOI:** 10.1186/s13063-018-3113-6

**Published:** 2019-01-07

**Authors:** Stewart J. Wiseman, Rozanna Meijboom, Maria del C. Valdés Hernández, Cyril Pernet, Eleni Sakka, Dominic Job, Adam D. Waldman, Joanna M. Wardlaw

**Affiliations:** 1 Edinburgh Imaging and Centre for Clinical Brain Sciences, University of Edinburgh, Edinburgh, UK; 2 UK Dementia Research Institute Edinburgh, University of Edinburgh, Edinburgh, UK; 3 CCBS, Chancellor’s Building, Royal Infirmary of Edinburgh, 49 Little France Crescent, Edinburgh, EH16 4SB UK

**Keywords:** Longitudinal, Multi-centre, Magnetic resonance imaging, Study design, Data sharing, Guidelines, Big data

## Abstract

**Background:**

Research involving brain imaging is important for understanding common brain diseases. Study endpoints can include features and measures derived from imaging modalities, providing a benchmark against which other phenotypical data can be assessed. In trials, imaging data provide objective evidence of beneficial and adverse outcomes. Multi-centre studies increase generalisability and statistical power. However, there is a lack of practical guidelines for the set-up and conduct of large neuroimaging studies.

**Methods:**

We address this deficit by describing aspects of study design and other essential practical considerations that will help researchers avoid common pitfalls and data loss.

**Results:**

The recommendations are grouped into seven categories: (1) planning, (2) defining the imaging endpoints, developing an imaging manual and managing the workflow, (3) performing a dummy run and testing the analysis methods, (4) acquiring the scans, (5) anonymising and transferring the data, (6) monitoring quality, and (7) using structured data and sharing data.

**Conclusions:**

Implementing these steps will lead to valuable and usable data and help to avoid imaging data wastage.

## Background

Research involving brain imaging contributes to our understanding of common brain diseases such as stroke [1], dementia [2], multiple sclerosis [3] and brain tumours [4]. Imaging endpoints are used in clinical trials [5, 6] to provide objective evidence of beneficial and adverse outcomes, and imaging-derived features have become phenotypes in their own right. Aims will differ across studies, and imaging can be used to drive main endpoints but also as an exploratory tool that contributes to our understanding of disease mechanisms. Longitudinal multi-centre studies, in which patients from different centres are imaged repeatedly over time, allow greater numbers of subjects to be recruited, and can generate insights into disease progression and outcomes that are not available in cross-sectional studies. Such studies are, however, expensive and complex to organise, and require careful co-ordination and management to optimise the answering of research hypotheses.

Large sample sizes help reduce uncertainty and increase reliability in clinical decisions and clinical practice strategies, which are then informed by research evidence. Multi-centre imaging studies are one response to the increasing demand for more data. Generalisability and statistical power are enhanced with a larger study sample size. Such studies promote collaborations across institutions and countries, and experts worldwide will have access to the large data sets and can combine their group expertise [7] (e.g. http://enigma.ini.usc.edu/).

It is important to ensure that appropriate technologies and strategies are used to manage the large amounts of data generated: some of the challenges include capturing, storing, analysing, searching, sharing, transferring, visualising, querying, protecting and updating data – all in addition to maintaining patient confidentiality and adhering to good clinical practice.

A carefully conducted longitudinal or multi-centre study will yield valuable data, which are essential for addressing the study’s research questions. It is unethical to conduct a study if there is a high risk of data loss or misuse, or other problems that restrict the ability to answer the research question. Not only are high-quality data essential for answering the predefined research question, these data could also help other scientists from all over the world answer other questions. Data sharing and open science are important contemporary topics in medical research. The International Committee of Medical Journal Editors, UK Research and Innovation (https://www.ukri.org/funding/information-for-award-holders/open-access/), and some of the charities now mandate data sharing [8]. This includes the computer code [9] (image processing pipelines) that generates the research output from images. *The Lancet* [10] is promoting improved management and sharing of research data as these reduce ‘research wastage’ and reward scientific diligence; see the REWARD Statement, http://www.thelancet.com/campaigns/efficiency/statement).

Guidelines for designing [11], recruiting to [12], organising [13] and performing quality assurance [14, 15] in multi-centre trials have been published. Brain imaging protocols for large multi-centre imaging data collections also exist, e.g. UK Biobank [16, 17], Alzheimer’s Disease Neuroimaging Initiative (http://adni.loni.usc.edu/methods/documents/mri-protocols/) and the Lothian Birth Cohort [18], and data standards for clinical research are supported by the Equator Network (http://www.equator-network.org/) and the National Institute of Neurological Disorders and Stroke (https://www.commondataelements.ninds.nih.gov/). Additionally, much emphasis has been placed on data sharing after primary studies are completed. A detailed report (http://www.humanbrainmapping.org/files/2016/COBIDASreport.pdf) – summarised in *Nature Neuroscience* [19] – by the Organization for Human Brain Mapping and its Committee on Best Practice in Data Analysis and Sharing (http://www.humanbrainmapping.org/cobidas) includes over 100 items to help plan, execute, report and share neuroimaging research. Recommendations already exist for multi-centre functional magnetic resonance imaging (fMRI) studies [20] that have specific guidance, such as how to organise metadata [21] from task-based fMRI. Methodological issues for guarding against false positives and overstating effect sizes in neurogenetic studies have been discussed [22]. However, concrete practical advice, in an easily digestible format, that guides the setting up of a longitudinal multi-centre structural neuroimaging study is lacking.

**Fig. 1:**
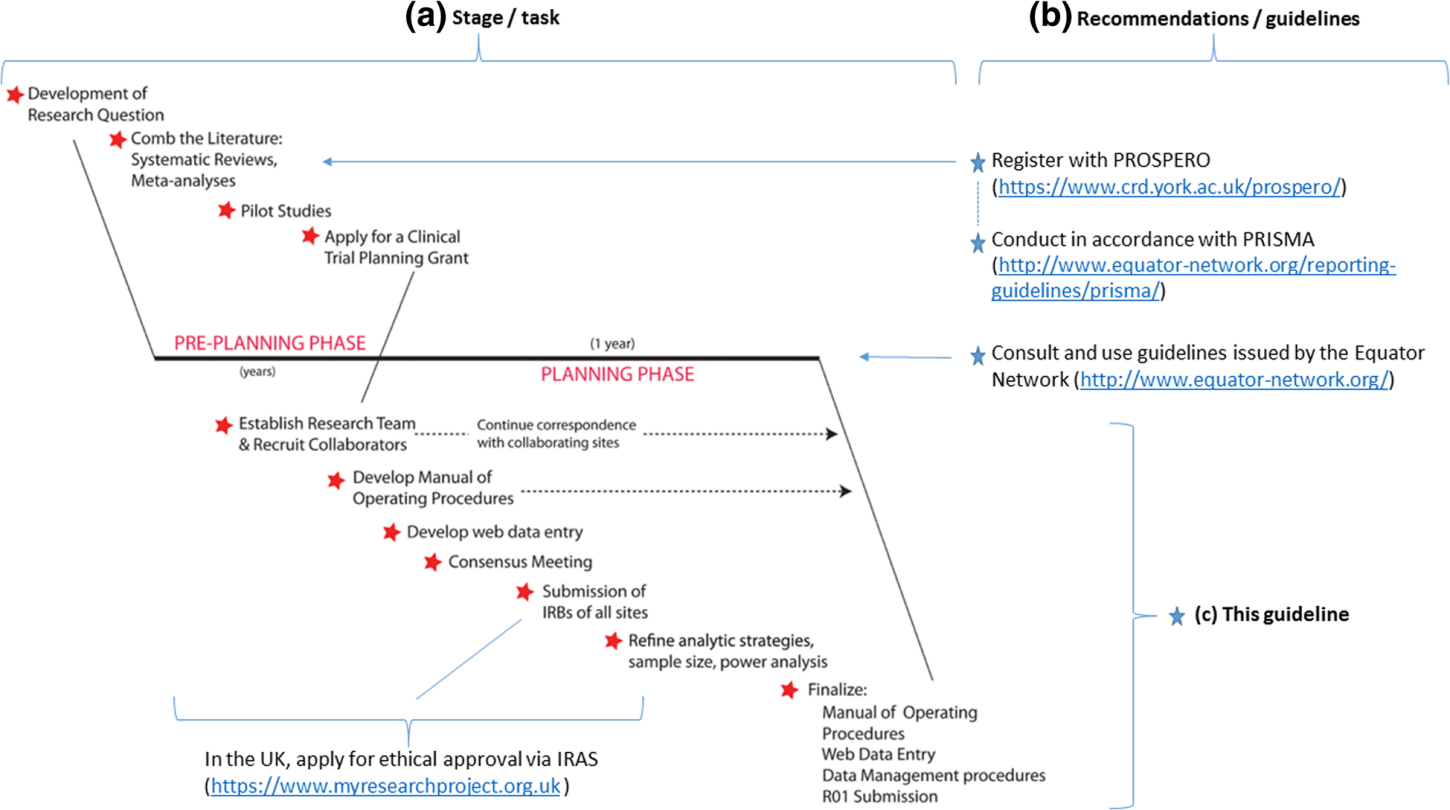
Where this guideline sits in the literature (adapted from Chung et al. [13]). IRAS Integrated Research Application System, IRB institutional review board

**Fig. 2:**
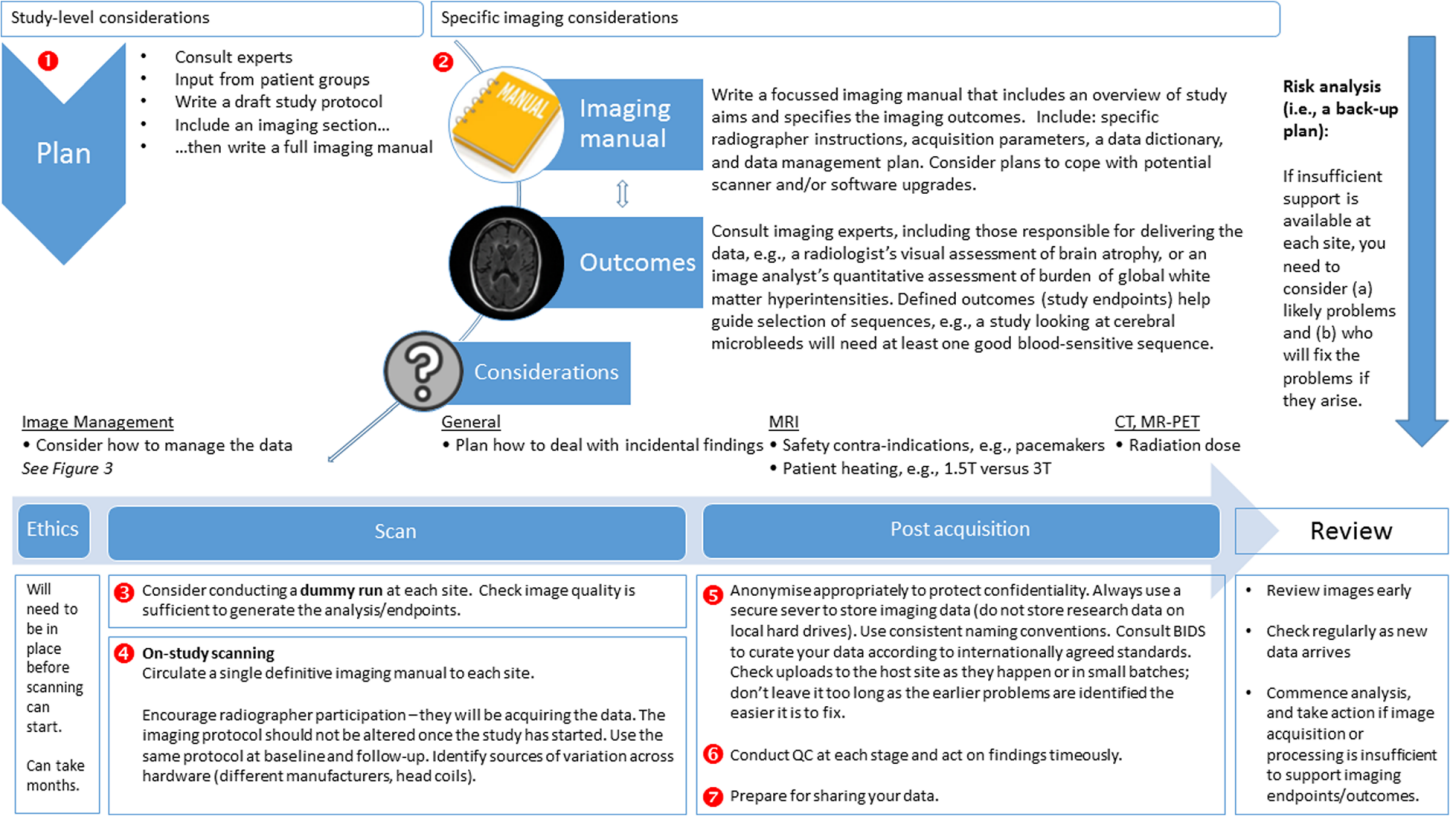
Summary of key points to consider in setting up a multi-centre brain imaging research study. BIDS brain imaging data structure, CT computed tomography, MRI magnetic resonance imaging, PET positron emission tomography, QC quality control

Here, we describe considerations that, in our experience, are necessary when designing and starting a longitudinal multi-centre brain imaging study. Figure 1 (adapted from Chung et al. [13]) shows where amongst the current recommendations and guidelines for conducting studies this work sits. This guideline contains specific technical examples and is illustrated with some common pitfalls that can threaten a study if not monitored and rectified when identified. Conducting studies appropriately will help achieve correct answers, avoid wastage of money and data, and ultimately improve patient care. A flow diagram of the experience-based recommended systematic approach we propose is illustrated in Fig. 2. Note that imaging should be considered at all stages in the design of a study, including the literature search, although we focus here on the more downstream aspects of study implementation since, in our experience, this is a neglected area.

## Results and discussion

### The key points explained

#### Planning phase

The early involvement of imaging personnel with relevant expertise cannot be overemphasised, since it will generate insights that can be fed into the main study protocol. These individuals are best placed to comment on what type of imaging will best answer the research question, for instance: which modality, if MRI the field strength and sequences, the image analysis methods, and who will carry out the analyses. Experts from the following fields bring a wealth of divergent knowledge:

- *Neuroradiologists* for general advice and insight, and for reading the images, reporting on incidental findings and advising on which sequences are best suited to answer the research question
- *Radiographers* for practical insights, e.g. a scan protocol with 25 min on-table will require a longer booking slot in a cohort of multiple sclerosis patients with severe movement difficulties
- *Medical physicists* to optimise technical parameters, e.g. to lower the specific absorption rate in MRI sequences with high flip angles or short repetition time, which is particularly relevant at 3 tesla, and for advice on protection from ionising radiation
- *Radiochemists* for advice on tracers for positron emission tomography (PET) and other radionuclide studies
- *Image analysts* to help specify the technical parameters to optimise efficient information extraction. For example, isotropic voxels can be used to reconstruct an image with the same in-plane resolution in different orientations but can bias diffusion estimates [23]; the planned analysis should guide the acquisition parameters
- *Data managers* for anonymising, storing accurately and robustly, and versioning metadata and moving large volumes of imaging data, which can be expensive and require specialists with specific knowledge of hardware and software
- *Facilities managers* to help cost and resource the project adequately, and to deal with unexpected costs, e.g. staff illness or equipment failure

#### The imaging manual and related systems of work

Writing the main study protocol correctly, and subsequently, an ethics application will be possible only after the planning and consultation phase. An imaging protocol should also be written and tested. It should include:

- Contact details (principal investigator and imaging staff)
- Brief introduction and study rationale
- Aims and hypotheses, clearly described
- Imaging endpoints and outputs—be specific, e.g. total brain tissue volume change over 1 year; white matter hyperintensity burden using the Fazekas scale, etc. This can be enhanced later based on new research findings, but at the minimum a framework that ensures the basic output needed to answer the research questions should be in place from the beginning
- Image acquisition including explicit acquisition parameters and radiographer instructions (e.g. “Do not angle acquisition direction on diffusion tensor imaging, but instead plan a straight axial with no in-plane rotation”); pictures can be helpful
- Anonymisation procedure, file naming conventions, upload procedure for Digital Imaging and Communications in Medicine files (DICOM)
- Quality control (QC) procedure for inter- and intra-site reliability and reproducibility
- Details of the image management system (see below)
- Appendices (further detailed technical parameters, log sheets to record notes specific to a scan session, e.g. ‘patient anxious’ or ‘only first two sequences acquired’)

**Fig. 3:**
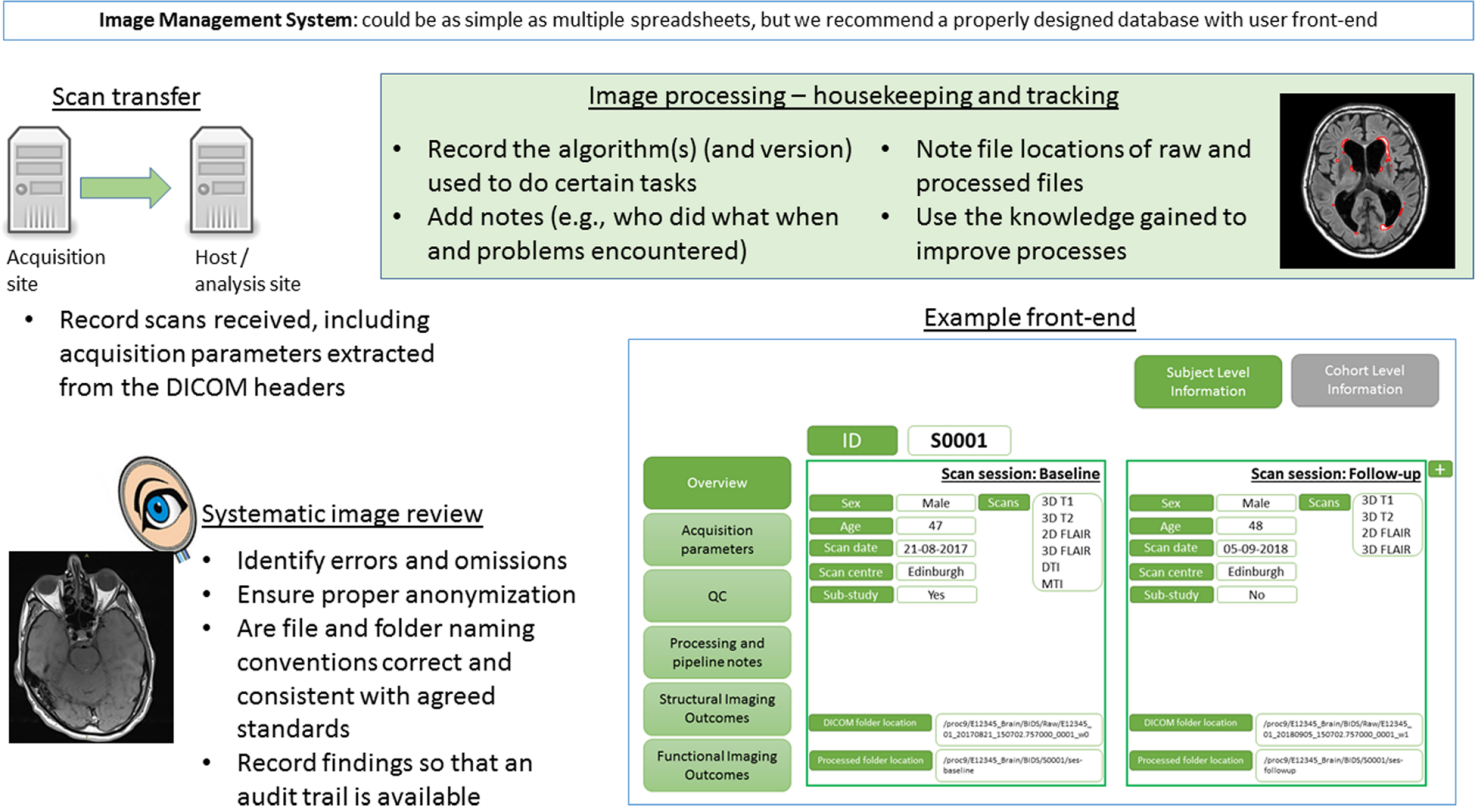
Image management system, including example screenshots of the user interface. DICOM Digital Imaging and Communications in Medicine (file type)

Once the imaging manual is live, it should not be altered. A change made after the study is underway will increase the noise in the data, make the analysis inefficient and potentially reduce the sample size if images cannot be used. However, it is also important to be pragmatic. The protocol should be altered if there is an essential improvement that is beneficial to the study. As an example, the authors implemented a new version of an imaging manual for a large multi-centre study to change the T2-weighted scan to a dual echo T2. This change permitted the generation of a proton density-weighted image, which allowed for improved brain extraction from the images. The same protocol (sequences and parameters) used at baseline should be used at the follow-up. Also consider:

- Incidental findings [24] and how they will be handled [25, 26], e.g. if studying multiple sclerosis, there should be a mechanism to deal with a suspected brain tumour. A cogent policy for management of incidental findings is now mandated in UK Research Ethics applications and by the Medical Research Council and Wellcome Trust, both major funders of human imaging research.
- If available, follow disease-specific agreed-upon recommendations that guide choice of scan sequences (see the example of a small vessel disease in [1]) and where relevant new methods of analysis (but ensure the proposed method is relevant to your population and research question and has been validated for your scans and patients).
- Harmonisation of sequences across sites: different sites might conduct imaging on different manufacturer’s hardware, and even the same scanner at two different sites might be running different software versions. It is important to investigate these issues and assess any impact on the proposed imaging outcome measures.
- A plan to cope with unforeseen circumstances, such as scanner upgrades.
- The availability of head coils should also be considered.
- In MRI studies, consider safety contra-indications (e.g. a cardiac pacemaker) and subject heating (specific absorption rate). Consider the radiation dose in studies using computed tomography, PET or any radionuclides.
- Consider how the image data and related phenotypes will be stored and managed. The recommendations presented in this paper require an image management system that will handle scan transfers, data housekeeping, systematic reviews of the data and data queries. Involving database experts that understand database schemas is the most robust approach. The upfront cost of developing such a system will be repaid in efficiency gains. Storage of, and linking to, other study data including genetic and clinical variables is a further step in integrating all study data. Combining neuroimaging data with other study variables, particularly if they include full genome sequencing, will result in massive datasets [27]. Figure 3 covers some elements of an image management system, including:
  - ◦ a record of the scan datasets received (when received, which centre sent the data and key variables extracted from the DICOM headers, such as scan date)
  - ◦ notes from the QC process (e.g. pass or fail, and if the scan failed, a reason)
  - ◦ links to where the raw data are stored, notes on how it was processed (including the software used and version numbers, problems encountered and who did the processing), links to where the processed output is stored

An image management system is important. The 3rd International Stroke Trial investigated alteplase in acute ischaemic stroke and used brain imaging as a critical component of the diagnosis and safety outcome assessments. Our centre coordinated imaging from 3035 patients in 156 centres in 12 countries from 2000 to 2012, yielding a total of over 7000 scans. In this trial, which used an efficient image management system, missing scans were queried in 4.2% of patients (only 17 of these (0.5%) were missing in the end) and 2.4% of scans had imaging issues rendering them unsuitable for reading. Either a commercial or open-source image management system is worth investigating. One example is the Hive database system (theHiveDB) [28], although we have not used it and are unable to comment on its utility. Researchers should attempt to minimise scan losses (by considering this guideline) but also allow for a percentage of losses in power calculations, just as for subject loss to follow-up.

#### Dummy run test scan

Testing the imaging protocol on a healthy volunteer or patient with the disease of interest is essential. Each site should do this and upload the dummy scan to the hosting facility to verify all aspects of the study are as expected (image quality, anonymisation, and upload and transfer procedures). On-study scanning should start only after acceptance. The analysis pipeline should also be tested at an early stage.

#### Acquire

High quality and consistent scans are essential for successful scientific studies. In some studies, images are assessed by radiologists or trained observers, who give their opinion on, for example, disease status or detailed visual scoring, and it is important that image quality is sufficient for such reads. Likewise, images are often processed using automated or semi-automated algorithms that require consistent contrast, resolution and subject positioning, which are dependent upon fixed acquisition parameters such as acquisition orientation, field of view, and matrix, to minimise between-scan noise.

##### Pitfalls

**Fig. 4:**
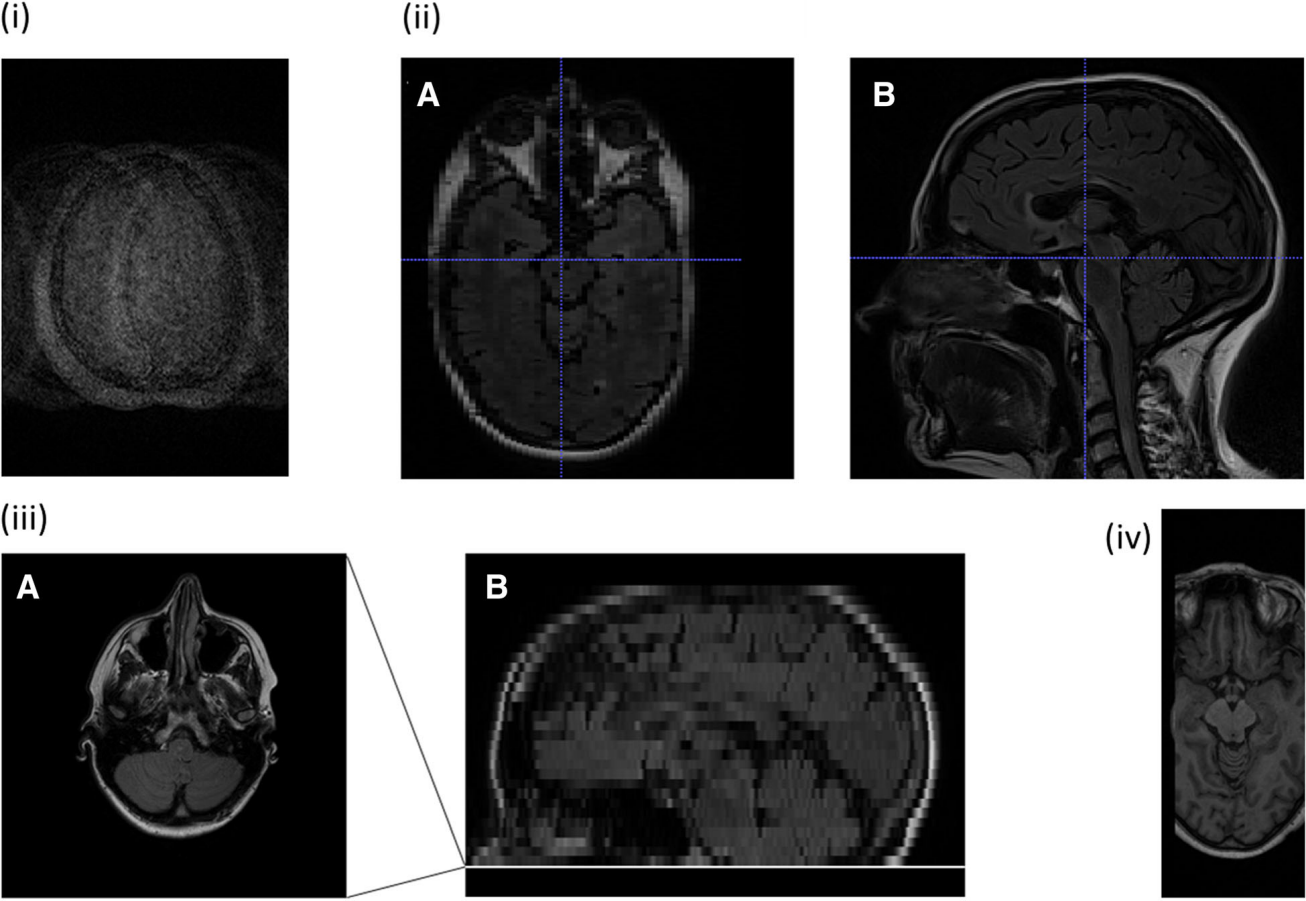
Examples of potentially useless scans: (a) Movement artefact resulting in an unusable scan. (b) MR brain scan with incorrect resolution in the axial plane (A) due to wrong acquisition plane (B) being used, potentially causing incorrect assessment of pathological features. (c) MR brain scan with insufficient brain coverage, resulting in a large portion of the cerebellum missed. The last slice was captured inferiorly in the axial plane (A), with corresponding level on the sagittal reconstruction for reference (B). (d) Incomplete transfer of the entire image acquisition, preventing complete image reconstruction and any analysis. MR magnetic resonance

Some subjects find it difficult to tolerate the scan session. Restless patients can affect scan quality, particularly for sequences like MRI fluid-attenuated inversion recovery (FLAIR) or susceptibility-weighted imaging, which are more sensitive to movement. Figure 4 has examples of some scan errors. Figure 4(a) shows an unusable FLAIR image due to subject movement. Here, time permitting, radiographers could ask the patient if they feel able to remain on the table while the scan is repeated. Alternatively, the protocol might permit a quick version of the scan, or use of motion correction with navigator echo methods, such as such as PROPELLER^1^ and BLADE.^2^ Other motion-correction tools also exist (for example, https://firmm.io/). If the problem is not rectified, it can be useful to append notes to an affected scan saying so, which prevents analysts from wasting time looking for better scans that do not exist. Movement artefacts are a known problem in imaging. Those used to working in medical health care rather than research should note that a subtle movement can affect automated processing algorithms, even in scans that are deemed satisfactory from a clinical perspective. Moreover, good quality scans are much more likely to be usable and re-usable for many different analyses, years after acquisition.

Figure 4(b) is an example of an image acquired (wrongly) in the sagittal direction, resulting in unacceptable in-plane resolution axially. To maximise information, it is important to scan the required anatomy fully and to a consistent standard that permits reproducible analysis. Figure 4(c) shows the last slice inferiorly from an axially acquired FLAIR brain scan with insufficient whole brain coverage, resulting in a large portion of the cerebellar tissue being missing. Not only does this reduce the chance of capturing all disease, but it means that the brain’s intracranial volume cannot be measured, an important metric for determining brain shrinkage.

Data quality and completeness should be checked at the end of the exam before the subject leaves the scanner, and problems should be rectified, time allowing.

#### Anonymise, file and folder names, and data upload and transfer

Data governance regulations require research data to be anonymised before leaving the health-care system. Ethics committees require anonymisation of personally identifiable information from the raw imaging DICOM data before it is sent to a central host site for analysis, particularly if outside the health provider environment. Participating imaging centres are usually diligent at removing patient names and unique identifiers, but it is also often important to anonymise sex, age and date of birth. The trial sponsor, Caldicott Guardian (https://www.igt.hscic.gov.uk/Caldicott2Principles.aspx) or another data controller may dictate what types of personally identifiable data are permitted in a particular study and this information should be in the imaging manual or the study protocol. The process by which subject-identifiable data are removed from the imaging dataset header files is important and must not also result in removal of data essential for subsequent image analysis. It is possible to recreate facial features, and hence identify individuals, from structural brain imaging data [29]. Consequently, it is recommend to de-face the images, and there are automated tools for this purpose (for example, https://surfer.nmr.mgh.harvard.edu/fswiki/mri_deface).

In longitudinal studies, it is crucial to be able to distinguish scans temporally. Thought should be given to the scan naming convention. The brain imaging data structure (BIDS) [30] (http://bids.neuroimaging.io/) recommends use of the “ses” prefix in studies with multiple time points to denote the session, e.g. “sub-001_ses-baseline” would indicate a baseline scan for the first subject while “sub-001_ses-12 months” would indicate a 12-month follow-up scan. Inclusion of the date in the scan file name can be helpful. Mixing up baseline and follow-up scans will lead to incorrect scientific results and confound the study.

Often in multi-centre imaging studies, a central site will be the main repository and analysis site. An incomplete upload of a source DICOM file from a satellite to the host site makes it impossible to reconstruct the full image, as shown in Fig. 4(d). Transfers should be checked on receipt for completeness as they happen, or in small batches while it is still possible to repeat the transfer before the original raw data are wiped from the scanner or archived by other means. Checking scans on arrival also allows systematic problems from a particular site to be identified early and rectified before more patients are scanned. It is poor practice to verify upload completeness after the data from many subjects have been transferred, since it makes the job of rectifying problems due to poor site performance or scan transfers more cumbersome if not impossible. Consider manual or automated scan checks. For example, if 60 image slices are expected, did 60 arrive? Regular checking identifies problems early before they multiply among many cases.

Transfers should be to a resilient and secure server infrastructure. Cloud-based or drop box methods that are in common use are unlikely to meet the security requirements.

#### Quality control

Brain imaging data needs to be processed to provide meaningful biomedical information. Such processing can be extensive, and processing pipelines are used to automate and facilitate this work and should have robust quality checks at each stage. As well as the examples in this paper, further examples exist, including details on what to look for, how to look for them and the causes (e.g. cbs.fas.harvard.edu/usr/mcmains/CBS_MRI_Qualitative_Quality_Control_Manual.pdf).

Here we describe the QC checks that should be used in brain imaging studies to verify accuracy, ordered by where in the flow of information from scan acquisition to imaging endpoint they should be conducted:

- *Image acquisition* – Radiographers should verify completeness (e.g. whole-brain coverage) and attempt to rectify movement and other artefacts at the time of acquisition, per the instructions in the protocol.
- *Data upload* – The person responsible (a radiographer, researcher or the data manager) should ensure proper anonymisation, then transfer the complete exam.
- *Data receipt* – The receiving centre should check that all expected images actually arrived.
- *Initial QC* – After the raw data have been converted to the processing format (e.g. NIfTI), the analyst needs to verify that the scans meet the prescribed quality. For example, was the correct field of view used? Are there any artefacts? Are any orientations not flipped? The analyst should also ensure folder and file naming conventions are consistent and meet the expectation of the processing pipeline.
- *Pre-processing QC* – The output should be checked. For example, did the brain extraction or defacing method perform adequately? Was there poor image registration?
- *Processing pipeline* – Check the compatibility of the output of each piece of software involved in the image processing pipeline beforehand on a subset of representative images from all centres involved.
- *Post-processing QC* – Check masks and manually edit them as required. The output files should be consistently named as per the agreed predefined conventions (e.g. S001_ses-baseline_acq-FLAIR_WMHvol).

QC is important as it can affect imaging endpoints, and hence, has the potential to confound a study. The Brain Development Cooperative Group reviewed the development of brain cortical thickness from 5 to 22 years of age and found that QC procedures had a significant impact on the assessment of cortical thickness developmental trajectories [31]. This finding has implications for other studies, in for example neurodegeneration, where including or excluding scan data based on how strictly the researcher applies QC procedures could influence the results. Ioannidis and colleagues [32] comment that *small effects can be difficult to distinguish from bias* and this applies equally to imaging research and thus, large sample sizes and high-quality data are critical. Large imaging studies may choose to use supervised machine learning in the QC pipeline to identify problematic images (e.g. UK Biobank [17], due to its sheer size). A visual inspection is usually preferable in smaller projects, since machine learning methods require large training sets. Automated and semi-automated systems for QC exist and should be used. They can provide a range of useful metrics, such as the signal-to-noise ratio. Some examples of tools and workflows include:

- http://preprocessed-connectomes-project.org/quality-assessment-protocol/
- https://poldracklab.github.io/mriqc/
- https://raamana.github.io/visualqc/readme.html

#### Structured data organisation and data sharing

BIDS [30] standardises the approach to describing and organising neuroimaging data. The use of a structured approach is critical, not only within a single site (so colleagues know what data they are dealing with) but also across collaborating sites, as it makes pooling data far simpler as scripts and processing pipelines do not need to be laboriously re-coded to cater for site-specific nuances. Additionally, the use of this standardised approach makes it more likely that other researchers will easily be able to work on the data, and there are an increasing number of software tools being developed that understand data organised according to BIDS, which opens the possibility for machine-readable analyses.

Collaborative neuroimaging data management systems that promote data sharing exist and their use is encouraged. Consider maximising the benefits of the research to society by making the data available to other researchers either through a university’s repository facilities or, if available and relevant, by submitting the data to a brain bank (for example, see the practical recommendations from the BRAINS Expert Working Group for enhancing the creation, use and reuse of neuroimaging data [33]).

## Conclusions

Imaging is an evolving science, and metrics derived from imaging are increasingly being used as study endpoints. We provide an overview of what is, from experience, essential for setting up a successful longitudinal or multi-centre imaging study. Following and implementing these steps will lead to valuable and usable data and help avoid wastage of imaging data and study failure.

1. Plan the study: This includes building relationships with imaging experts who are best placed to ensure the study meets its aims.
2. Set up an imaging manual with the essential details of the imaging process (e.g. include the scanning protocol and analysis methods). Do this before you apply for a grant or ethical approval. Implement an image management system.
3. Perform a dummy run before the trial starts in which you test your scan protocol on a volunteer and go through the other imaging procedures needed for successful study performance. Test your analysis methods.
4. Carefully acquire scans of high quality that are consistent across the study.
5. Monitor the scan uploading process and make sure the scans are received as expected at the central data site. Decide how to anonymise scans before transferal to other sites.
6. Implement data QC and quality assurance and feedback at all appropriate study steps (image acquisition, data upload, pre-processing and post-processing) to identify problems early, to give feedback to correct problems and thus, to ensure you have high-quality data.
7. Use structured data and share the data.

## Abbreviations

BIDS: Brain imaging data structure
CT: Computed tomography
DICOM: Digital Imaging and Communications in Medicine (file type)
FLAIR: Fluid-attenuated inversion recovery
fMRI: Functional MRI
IRAS: Integrated Research Application System
IRB: Institutional review board
MRI: Magnetic resonance imaging
PET: Positron emission tomography
QC: Quality control

## Footnotes

1 GE (http://www3.gehealthcare.com/en/products/categories/magnetic_resonance_imaging/mr_applications/body_imaging/propeller)

2 Siemens (https://www.healthcare.siemens.co.uk/magnetic-resonance-imaging/options-and-upgrades/clinical-applications/syngo-blade/features-benefits)
